# *Mycoplasma pneumoniae*-Induced-Stevens Johnson Syndrome: Rare Occurrence in an Adult Patient

**DOI:** 10.1155/2012/430490

**Published:** 2012-08-16

**Authors:** Samad Rasul, Faria Farhat, Yared Endailalu, Fatima Tabassum Khan, Vishal Poddar

**Affiliations:** ^1^Division of Infectious Diseases, Department of Internal Medicine, Howard University Hospital, 2041 Georgia Avenue NW, Washington, DC 20060, USA; ^2^Department of Internal Medicine, Howard University Hospital, 2041 Georgia Avenue NW, Washington, DC 20060, USA; ^3^Division of Pulmonary, Department of Internal Medicine, Howard University Hospital, 2041 Georgia Avenue NW, Washington, DC 20060, USA

## Abstract

Stevens-Johnson syndrome (SJS) is an uncommon occurrence in *Mycoplasma pneumoniae* (*M. pneumoniae*) infection (1–5%) and has been mainly reported in children and young adults. We present a case of SJS in a 32-year-old male induced by *M. pneumoniae* infection. This patient presented with fever, cough, and massive occupation of mucus membranes with swelling, erythema, and necrosis accompanied by a generalized cutaneous rash. He clinically responded after treatment with antibiotics and IVIG. SJS is usually a drug-induced condition; however, *M. pneumoniae* is the commonest infectious cause and should be considered in the differential diagnosis.

## 1. Case Report

 A 32-year-old African American male presented to our hospital with a generalized skin rash for the past 3 days. He developed a productive cough, sore throat, and fever one week prior to developing this rash. He also noted rhinorrhea and pink eyes with visual blurring and was prescribed erythromycin eye ointment by a physician. The rash appeared one day using the ointment and was described as a nonpruritic, painless eruption of “bumps” starting on his back and rapidly spreading over his chest and hands. The rash progressed over 3 days and he experienced swelling of both eyelids along with blistering and crusting of his lips. He also experienced mild dysphagia and decided to come to the hospital for further evaluation. He admitted to being allergic to sulfa drugs; however, he had not received any antibiotics other than the erythromycin ointment. He denied any travel, animal exposure, or insect bites. He also denied having any sexual encounters in recent weeks. He had a history of occasional tobacco and marijuana usage, and his last use had been over 6 months ago.

**Figure 1:**
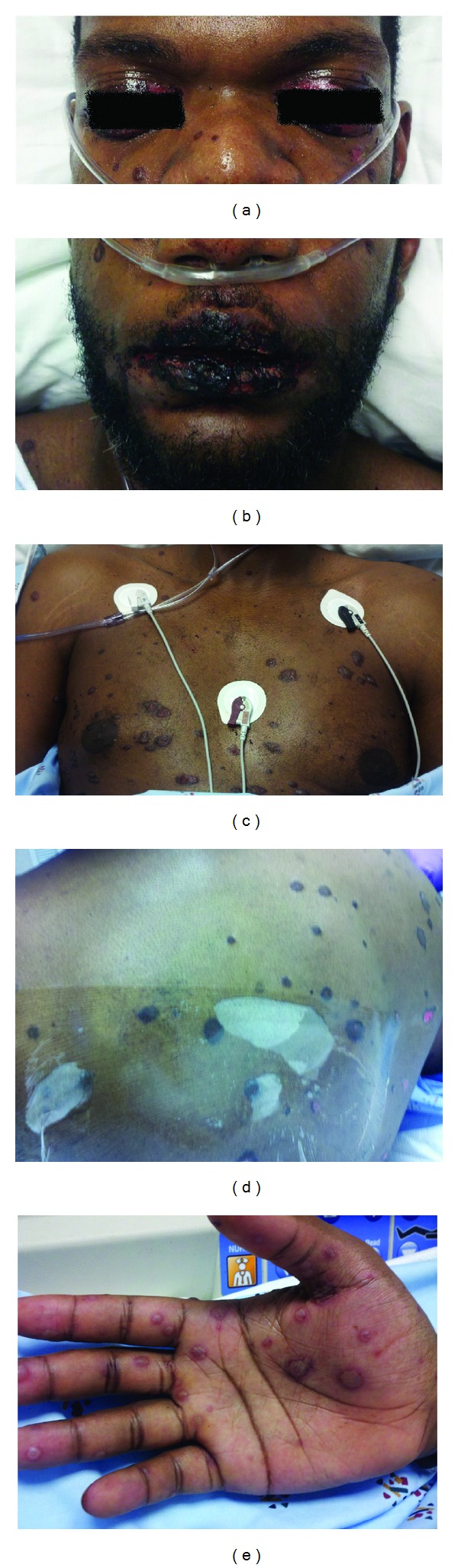
(a) Swelling and necrosis of eyelids with conjunctival erythema. (b) Severe necrosis of oral mucosa. (c) Macules and patches on chest. (d) Rash on back with areas of necrosis and denudation. (e) Targetoid lesions on right palm.

On physical examination, he had a low-grade fever of 100.5 Fahrenheit, his pulse rate was 90 beats per minute, respiratory rate was 20 breaths per minute, blood pressure was 130/70 mm of Hg, and he was saturating 90% on room air. His upper and lower eyelids were swollen bilaterally and he had obvious conjunctival erythema (Figure 1(a)). There were marked necrosis and mucosal sloughing involving his lips (Figure 1(b)). He had generalized macules and patches on his chest and back (Figures 1(c) and 1(d)), which to a lesser extent also involved his face, abdomen, and arms. The rash appeared in different stages of healing, and targetoid lesions were noted on selected sites most notably his palms (Figure 1(e)). His genitalia and lower extremities were not involved. On pulmonary examination, the right lower lung fields had fine end-inspiratory rales.

**Figure 2:**
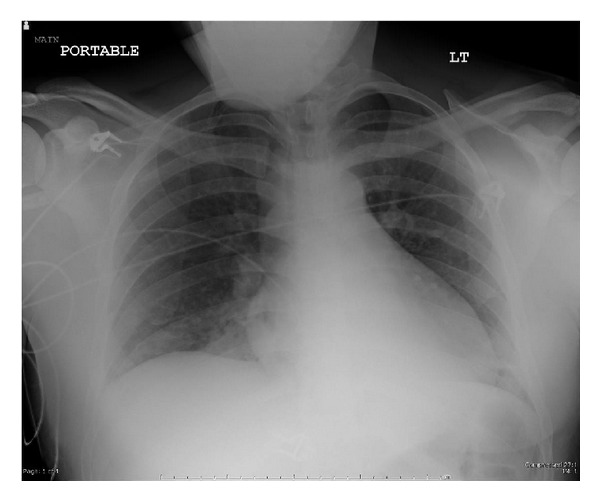
Right lower lung infiltrate on chest X-ray.

Pertinent laboratory results (with reference values in parenthesis) were as follows: serum creatinine 1.1 mg/dL (0.7–1.4 mg/dL), total bilirubin 0.9 mg/dL (0.2–1.2 mg/dL), AST 99 IU/L (0–50 IU/L), ALT 31 IU/L (0–55 IU/L), alkaline phosphatase 37 IU/L (30–165 IU/L), albumin 3.1 grams/dL (3.2–5.5 grams/dL), total WBC count 11.6 cells/mm^3^ (3.2–10.6 cells/mm^3^), hemoglobin 13.3 grams/dL (12.1–15.9 grams/dL), and platelet count 228 cells/mm^3^ (175–400 cells/mm^3^). A urine toxicology screen was obtained and returned negative. His chest X-ray revealed a right lower lobe infiltrate (Figure 2). Blood and urine cultures were negative. In view of characteristic skin rash with mucosal and conjunctival involvement, a diagnosis of Stevens-Johnson syndrome was considered. He was started on moxifloxacin and received methylprednisolone 80 mg IV for a total of 4 doses with minimal response. He was then given 1 g/kg intravenous immunoglobulin (IVIG) divided in to 3 doses, administered on consecutive days while continuing supportive care. Skin and mucus membrane lesions started to heal within 48 hours of IVIG.

**Figure 3:**
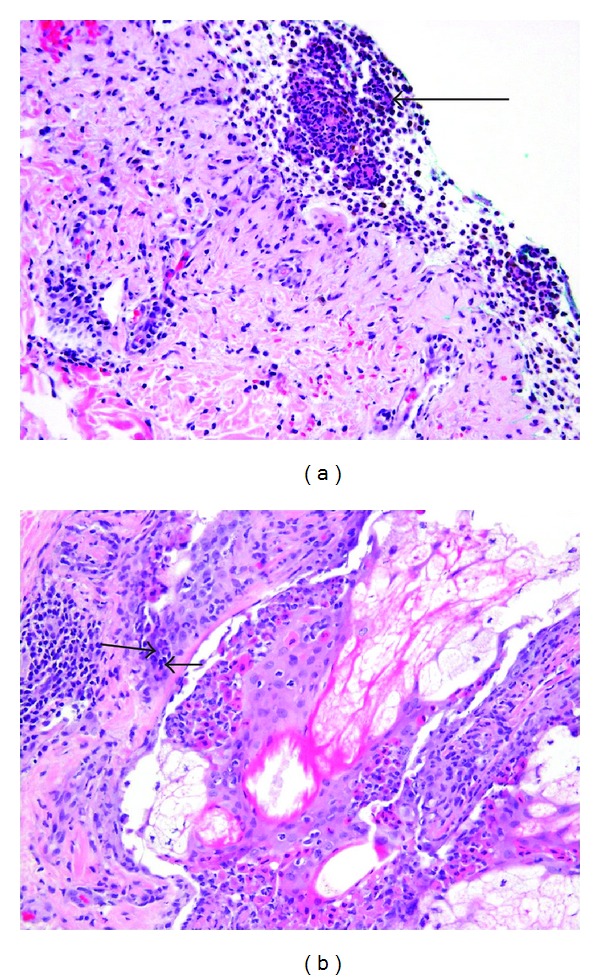
(a) Subepithelial abscess (arrow) on skin biopsy from left arm. (b) Necrotic infundibular epithelium with necrotic keratinocytes (arrows) on skin biopsy.

A workup to determine the underlying etiology was pursued while supportive management was continued. Tests for syphilis, Epstein-Barr virus, Herpes simplex virus, *Chlamydophila pneumoniae*, hepatitis B and C viruses, influenza A/B viruses, and human immunodeficiency virus (HIV) returned negative. A punch biopsy of skin from his left arm was performed and revealed subepidermal inflammation with necrotic infundibular epithelium and necrotic keratinocytes (Figures 3(a) and 3(b)) consistent with Stevens-Johnson syndrome. On day 5 of his hospital course, *Mycoplasma pneumoniae* IgM by enzyme immunoassay (EIA) returned at a level of 3010 U/mL (<770 U/mL) with *Mycoplasma pneumoniae* IgG > 5.00 ISR (≤0.90 ISR negative, 0.91–1.09 ISR equivocal, ≥1.10 ISR positive). We thus concluded that this was a case of Stevens-Johnson syndrome secondary to *Mycoplasma pneumoniae* infection.

## 2. Discussion

*Mycoplasma pneumoniae* is one of the commonest causes of atypical pneumonia. 25–33% of infected patients may show some cutaneous manifestations most notably exanthems, urticaria, and SJS [1]. Infection with *M. pneumoniae* may produce a variety of extrapulmonary manifestations, including neurological, hepatic, cardiac, and dermatological diseases before, during, or after pulmonary involvement [2]. SJS is a critical disease, characterized by high fever, extensive blistering lesions, mucosal involvement, and atypical target-like skin lesions which often progress to toxic epidermal necrolysis (TEN) [2]. In addition to SJS, *M. pneumoniae* has also been noted to cause erythema multiforme (EM) and “atypical SJS” manifesting as severe mucositis without skin lesions [3]. The distinction between SJS and EM major is controversial; however mucous membrane involvement generally favors a diagnosis of SJS [3]. SJS usually evolves as an idiosyncratic reaction to medication (typically anticonvulsants or antibiotics), but *M. pneumoniae *has been cited as the most common infectious cause [3]. Many other infectious agents have been known to cause SJS, but none as commonly as *M. pneumoniae *[4].

*M. pneumoniae*-associated SJS most commonly affects children and young adults (mean age 15.3 years to 19 years) compared with drug associated SJS (mean age 31.4 years to 46.6 years) [2, 3]. In younger patients, SJS may be preceded by symptoms of upper respiratory tract infection from 2 days to 2 weeks before the appearance of the rash [5]. Most adults generally develop symptoms and rash almost simultaneously, perhaps due to an immune reaction to previous exposure [2]. Oral lesions are present in 100% of cases, genital lesions in 75%, and ocular lesions in 66% [1]. *M. pneumonia*-induced SJS is associated with less severe complications and less internal organ involvement than those resulting from other causes [2, 3]. Pneumonia is more commonly associated with drug-induced as opposed to *M. pneumoniae*-associated SJS [3].

Patients with *M. pneumonia*-associated SJS frequently receive antibiotics early during their illness before cutaneous involvement which may be a confounding factor in determining the precise etiology of SJS [3, 6]. *M. pneumoniae *has occasionally been isolated from the involved skin of effected patients; however, these lesions are most commonly believed to result from immune complex-mediated vascular injury, autoimmune reaction, and cell-mediated immune response [6]. The diagnosis of SJS is clinical, and a detailed history should be obtained to identify and discontinue any potential causative drugs. A skin biopsy should be performed in all cases to exclude other conditions. *M. pneumoniae *work up should be considered in patients who present with SJS, particularly if there is no history of recent medication use [3].

The laboratory diagnosis of *Mycoplasma* infections can be obtained by culture, serology utilizing enzyme immunoassay (EIA), or complement fixation (CF) methods and polymerase chain reaction (PCR) based testing. Isolation of *M. pneumoniae* by culture is considered to be the gold standard of diagnosis, but requires 10–14 days and is not readable available [7]. Serology is most frequently used, and the most widely available methods are the CF and EIA tests [7]. The CF IgG antibody usually peaks 1–3 weeks after the onset of infection, and a fourfold increase in CF antibody titer is felt to be diagnostic [7]. EIA-based-tests that detect both IgM and IgG are more sensitive and specific than CF; however, the production of IgM antibodies may be inconsistent during the acute phase of infection, particularly in adults [7]. A high IgM antibody level by EIA method in our patient in the second week of illness strongly suggests that this was a case of acute *Mycoplasma pneumoniae* infection. PCR-based methods have gained popularity but are less sensitive compared to serological tests [8]. Some authors recommended using serology and PCR in parallel to diagnose *Mycoplasma* infection [8].

The management of *M. pneumoniae-*associated SJS includes supportive care with special attention to fluid and electrolyte balance [3, 9]. Antibiotics have been used mostly for SJS due to *M. pneumoniae* infection or cases with secondary complications or sepsis [3]. The role of corticosteroids in the treatment of SJS is generally controversial due to concern for secondary infections [3]. However, recently published data from an observational study noted a possible beneficial effect in patients treated with corticosteroids [9]. IVIG (total dose of 3 g/kg in divided doses over 3 days) may be considered in severe cases [3]. One observational study found significant benefit of using IVIG in children with drug-induced SJS [10]. These findings have not been supported by others [11]. Evidence for IVIG use in adult patients is lacking. Sepsis or severe organ involvement has rarely been reported in *M. pneumonia*-associated SJS [3]. Younger patients are known to have a shorter course of hospital stay (<30 days) compared to adults [2]. The most common long-term complication is ocular involvement. Ophthalmology evaluation is recommended both at the time of diagnosis and prior to hospital discharge [2, 3].

We believe that this case is unique in a few aspects. (1) *M. pneumoniae* associated SJS is more common in children and young adults <21 years of age and is unusual in patients above this age range. (2) Our patient developed URI and conjunctival symptoms 1 week before skin lesions and mucositis, which is an atypical lag period in adults. (3) Our patient had clinical and radiological evidence of pneumonia, which is less common in *M pneumonia*-associated SJS. (4) Our patient had high levels of *Mycoplasma pneumoniae* IgM antibodies which is not common for primary infections or reexposure in adults. Based on these findings, we suggest that *M. pneumoniae* infections can have an atypical presentation in adults and should be considered in the differential diagnosis of SJS in the appropriate clinical setting.
